# Mental health disorders, functioning and health-related quality of life among extensively hospitalized patients due to severe self-harm – results from the Extreme Challenges project

**DOI:** 10.3389/fpsyt.2023.1258025

**Published:** 2023-10-18

**Authors:** Tuva Langjord, Geir Pedersen, Tone Bovim, Tore Buer Christensen, Ingeborg Ulltveit-Moe Eikenæs, Oddbjørn Hove, Arvid Nikolai Kildahl, Erlend Mork, Astrid Berge Norheim, Ruth Kari Ramleth, Petter Andreas Ringen, Kristin Lie Romm, Johan Siqveland, Thea Schønning, Line Stänicke, Terje Torgersen, Mona Pettersen, Tone Tveit, Øyvind Urnes, Fredrik Walby, Elfrida Hartveit Kvarstein

**Affiliations:** ^1^Section for Personality Psychiatry and Specialized Treatments, Department for National and Regional Functions, Division of Mental Health and Addiction, Oslo University Hospital, Oslo, Norway; ^2^Faculty of Social Sciences, Department of Psychology, University of Oslo, Oslo, Norway; ^3^Network for Personality Disorders, Section for Personality Psychiatry and Specialized Treatments, Department for National and Regional Functions, Division of Mental Health and Addiction, Oslo University Hospital, Oslo, Norway; ^4^Institute of Basic Medical Sciences, University of Oslo, Oslo, Norway; ^5^Regional Centre – Violence, Trauma and Suicide Prevention, Oslo, Norway; ^6^Department of Acute Medicine, Oslo University Hospital, Oslo, Norway; ^7^Hospital of Southern Norway, Arendal, Norway; ^8^National Advisory Unit Personality Psychiatry, Section for Personality Psychiatry and Specialized Treatments, Department for National and Regional Functions, Division of Mental Health and Addiction, Oslo University Hospital, Oslo, Norway; ^9^Department of Research and Innovation, Helse Fonna Health Trust, Haugesund, Norway; ^10^Norwegian Centre of Competence for Intellectual Disabilities and Mental Health, Department for National and Regional Functions, Division of Mental Health and Addiction, Oslo University Hospital, Oslo, Norway; ^11^Nevsom Norwegian Centre of Expertise for Neurodevelopmental Disorders and Hypersomnias, Oslo University Hospital, Oslo, Norway; ^12^Early Intervention in Psychosis Advisory Unit for Southeast Norway, Division of Mental Health and Addiction, Oslo University Hospital, Oslo, Norway; ^13^Diakonhjemmet Hospital, Oslo, Norway; ^14^Department for Child and Adolescent Psychiatry, Oslo University Hospital, Oslo, Norway; ^15^National Centre for Suicide Research and Prevention, Institute of Clinical Medicine, University of Oslo, Oslo, Norway; ^16^Division of Mental Health and Addiction, Oslo University Hospital, Oslo, Norway; ^17^Institute of Clinical Medicine, University of Oslo, Oslo, Norway; ^18^Department for Research, Division of Mental Health and Addiction, Akershus University Hospital, Oslo, Norway; ^19^Faculty of Medicine, University of Oslo, Oslo, Norway; ^20^Child and Adolescent Psychiatry, Nic Waal Institute, Lovisenberg Hospital, Oslo, Norway; ^21^Department of Mental Health Care, St. Olavs Hospital, Trondheim, Norway; ^22^Department of Mental Health, Faculty of Medicine and Health Sciences, Norwegian University of Science and Technology, Trondheim, Norway; ^23^Department of Health and Care Sciences, UiT The Arctic University of Norway, Tromsø, Norway; ^24^Division of Mental Health and Addiction, Bergen University Hospital, Bergen, Norway

**Keywords:** self-harm, psychopathology, mental health disorder, hospitalization, inpatient

## Abstract

**Background:**

Severe self-harm leading to extensive hospitalization generates extreme challenges for patients, families, and health services. Controversies regarding diagnoses and health care often follow. Most evidence-based treatments targeting self-harm are designed for borderline personality disorder (BPD). However, current knowledge about mental health status among individuals with severe self-harm is limited.

**Objectives:**

To investigate psychopathology among patients extensively hospitalized due to severe or frequent self-harming behaviors.

**Method:**

A cross sectional study (period 2019–2021) targeting psychiatric inpatients (>18 years) with frequent (>5) or long (>4 weeks) admissions last year due to self-harm. The target sample (*N* = 42, from 12 hospitals across all Norwegian health regions) was compared to individuals admitted to outpatient personality disorder (PD) treatment within specialist mental health services in the same period (*N* = 389). Clinicians performed interviews on self-harm and psychopathology, supplemented by self-report.

**Results:**

The target sample were young adults, mainly female, with considerable hospitalization and self-harming behaviors, both significantly more extensive than the comparison group. The majority in both groups reported self-harm onset <18 years. The target sample reported increasing severity of self-harm acts and suicidal intention over time. Both samples had high levels of childhood trauma, impaired personality functioning, and a majority fulfilled criteria for PD. In the target sample, comorbid depression, PTSD, anxiety disorders, and substance use occurred more frequently and in 50%, psychosis/dissociative disorder/autism spectrum disorder/ADHD was reported (outpatient comparison sample: 9%). 35% in the target sample screened over cut-off for possible intellectual disability. The target sample reported poor psychosocial functioning and health-related quality of life – greater impairment than the outpatient comparison sample.

**Conclusion:**

The study reveals that severe self-harm inpatients have complex psychopathology and highlights the importance of individualized and thorough assessment among patients with severe and/or repetitive self-harm.

## 1. Introduction

Self-harming behaviors are common but have varying severity and frequency, and prevalence has increased the past decades (1). Among adults (>18 years) prevalence rates of self-harm range 4–23% (2, 3) – the highest rates found in the age-group 20–24 years (4). Similar tendencies are reported in studies of young British women [19.7%, (5)], Norwegian adolescents [16.2%, (1)], and Norwegian university students [19.6%, (6)]. Although the most severe self-harm conditions are less common, they are more prevalent in adolescent and younger adult populations (7). High repetition self-harm can be linked to long-standing psychosocial vulnerabilities (8). The subgroup of severely self-harming individuals has received little systematic attention, and there is a lack of studies focusing on extreme situations. As one of few, if any, the present study investigates a severely challenged cohort with frequent and/or lengthy psychiatric hospital admissions due to self-harming.

A former interview-based screening investigation in Norwegian psychiatric hospitals (response rate 74%) confirmed that extensive psychiatric hospitalization due to persisting, severe self-harm risk was present in all four health regions with a mean of 2 patients per inpatient department during the last year (9). The study further indicated considerable health threat and noteworthy mortality risk among inpatients with substantial self-harming behaviors. Severe medical sequelae were reported in approximately one fourth of the inpatients, and within the one-year investigation period, five patients died (1%). Disagreement among health professionals and uncertainty concerning patients’ mental health disorder led to major collaboration problems which affected the patients’ treatment. The study emphasized that such situations are highly challenging for both patients, families, and services, and recommended further research investigating psychopathology in the severely self-harming cohort.

DSM-5 defines non-suicidal self-injury (NSSI) as “deliberate, physical, self-harming behavior, but no suicide intent” [DSM-5 (10)] and includes skin cutting, hitting self, burning, stabbing, picking wounds and other tissue damage, whereas deliberate self-ham (DSH) often also includes intoxications and suicidal acts (11, 12). Extreme self-harming behaviors such as swallowing batteries, amputations or non-sterile injections are described in both medical and mental health settings (13). Self-harm can thus entail high degree of injury and also lethality. Self-harm (SH) will in our study refer to self-harming behavior regardless of intent.

Self-harm is a key risk factor for later suicide (14–16), increasingly so with repeated self-harm (8), and self-harm with subsequent medical attention has been identified as the most effective predictor of suicide behaviors (17). In many cases, there may be oscillation between self-harm and suicidal intention, or the behaviors may entail severe risk for health and life. Suicidal or non-suicidal intent may thus be hard to discern. The current study focuses on individuals hospitalized lengthy and/or frequently due to any kind of self-harming behavior. Self-harm in the context of this study will therefore refer to self-injury regardless of severity, suicidal ideation and attempts, and in line with Klonsky et al. (18) and Sadath et al. (19) include high risk behaviors such as intoxications, severe cutting, strangulation and similar.

Self-harm is associated with poor mental health, and impaired personality functioning is a central aspect (20, 21). A broad range of personality disorder (PD) traits have been identified as predictors of self-harm. These include emotional dysregulation, impulsivity, negative affectivity, hostility as well as introversion, and detachment (22). There is a consistent and large association between PDs and repetition of self-harm (8). Borderline personality disorder (BPD), by definition characterized by self-harm and suicidal features, has been specifically associated with high rates of hospitalization (23, 24). However, self-harming behaviors are also described among individuals with other mental disorders, such as post-traumatic stress disorder [PTSD (25)], eating disorders (26), schizophrenia (27), and neurodevelopment disorders such as autism spectrum disorder [ASD; (28)], intellectual disabilities (29), and borderline intellectual functioning (30). This broader picture is illustrated by the finding that adolescents who engage in frequent self-harm have an increased likelihood of any hospitalization as well as having mood disorders, PDs and PTSD (31). Furthermore, comorbidity of disorders is often associated with poorer functioning, more prevailing conditions, (32–34) and in a recent study, also with repeated self-harm (19). Thus, while evidence-based treatment interventions targeting self-harm are often designed for BPD, relevant research highlights larger diagnostic variation and in severe cases, comorbidity may further complicate the situation. Better understanding of mental disorder and complex comorbidity in a severely self-harming population may have high relevance for the choice and design of treatment approach, both therapeutic intervention, rehabilitation, and mobilization of support systems.

The lack of knowledge about what characterizes individuals with severe self-harm highlights the need for further investigation. A cross-sectional research collaboration, “Extreme Challenges”; involving psychiatric hospitals situated in all four health regions of Norway, was established for this purpose. It targets a subgroup of patients with current, extensive psychiatric hospitalization due to self-harming behaviors. A multisite design was chosen as the target sample was likely to be rare. As a whole, this research project aims for better understanding of the challenges related to the severe self-harming population. It includes exploration of: (1) Patients’ mental health disorder status, (2) Health service utilization, (3) Collaborations during inpatient treatment and across services, (4) Qualitative investigation of patients’ experiences, and (5) Transdiagnostic perspectives on emotional dysregulation and specific aspects of personality functioning.

The present study provides a broad diagnostic evaluation and aims to investigate (1) mental health disorder status, (2) levels of functioning and health-related quality of life, and (3) compare the target sample of inpatients with a cross regional sample admitted to specialized outpatient PD treatment. In this comparison we hypothesized the following: (1) Self-harm was more severe and started at an earlier age in the target sample; (2) The target sample had more severe personality disorder and higher mental health disorder comorbidity; (3) The target sample had poorer social functioning and poorer health-related quality of life.

## 2. Materials and methods

### 2.1. Overall study design

The present study has a quantitative, cross sectional, multisite design. The data collection was developed in cooperation with a national project group (clinicians, researchers, and patient and public involvement (PPI) representatives). This group was established in 2016 as a response to repeated referrals to a national advisory unit for PD assessment and treatment, from clinicians in psychiatric hospitals concerning complex, high-risk clinical situations of repeated and severe self-harm (SH) or suicide attempts (SA). For outpatient comparison, the project used cross sectional data retrieved from the quality register of the Norwegian Network for Personality disorders (35). The Network is an ongoing, well-established cross regional clinical research collaboration including outpatient units providing PD treatment on a specialist mental health service level.

### 2.2. The target sample (TS) – data collection and participants

The consequence of SH/SA in terms of psychiatric hospitalization was targeted in this study and inclusion criteria for TS were patients with frequent psychiatric hospital stays the last 12 months (defined as >5 admissions) and/or long hospital stays (>4 weeks duration) due to severe SH/SA or the risk thereof, irrespective of frequency, type or severity of SH/SA. The definition was developed by consensus within the national project group and used in the preceding screening.

The TS setting was limited to inpatient units within adult psychiatry (age 18–65 years). All health trusts in each of the four health regions of Norway were invited to participate (Supplementary Table 1a). Medical/surgical departments were not included in this study nor highly specialized clinics within mental health such as geriatrics, substance use/addiction, eating disorders, developmental disorders, or intellectual disability.

Recruitment of hospitals was facilitated by members of the national project group, information at relevant national conferences, written information with formal invitations by e-mails to relevant hospitals and information meetings arranged at local hospitals. Clinicians in the participating hospitals identified eligible patients, invited them to participate and upon patient consent, administrated self-reports and interviews.

Systematic training for the clinicians was provided in 2019–2021 in collaboration with experts in the national project group. The training included: (1) All participating clinicians were invited to workshops on diagnostic interviews and assessments arranged locally by the project leaders. Participation was optional. (2) To ensure availability of training, relevant information was also shared on a project website in Norwegian language throughout the investigation period.

TS consisted of 42 patients from 12 hospitals representing all health regions in Norway (Supplementary Table 1a). The data collection was October 2019–June 2021.

### 2.3. The comparison sample (CS) – data collection and participants

Data for CS from the Network for Personality Disorders quality register included all individuals who had been referred and considered eligible for specialized PD treatment in the period 2019–2020.

Further description of the treatment units, data collection and the provision of regular clinician training courses on diagnostic assessments and self-report instruments in the Network is presented in two recent publications with data from corresponding periods (35–37).

CS constituted 389 patients from 13 different treatment units across Norway, the majority from south-eastern and western regions (Supplementary Tables 1a,b).

### 2.4. Assessment procedures

Assessments and interviews in TS were designed to enable comparison to CS and provide more detailed exploration of TS. The total TS assessment package covered all five foci of the Extreme Challenges research project (Supplementary Table 2). To lessen the burden for poorly functioning individuals, patient self-report and clinician administered interviews/reports in TS was divided in two separate packages, the first enquiring about the target situation (SH/SA and treatment, current hospital and former treatment), the second the diagnostic evaluation, symptoms and functioning. Patient self-report was completed during the hospital stay. Clinician assessments concerning the target situation were also completed during the hospital stay. For patients with short admissions the full diagnostic assessment (clinician interviews) was completed after discharge.

All CS data was retrieved from the quality register and represented the regular patient assessment administered within the outpatient units before starting treatment. Assessments were performed over 3–5 outpatient sessions.

### 2.5. Assessments/measures administered in TS and CS

1. Self-harm (SH) and suicide attempts (SA) assessed by patient self-report was based on items specially designed for the Network recording lifetime (ever) and last 6 months (answer options: yes/no, and if yes; specified categories of frequency and age first time). Description of SH/SA patient-report is given in a Supplementary Table 3).
2. Former hospital admissions and other former outpatient treatment was assessed by patient self-report based on items specially designed for the Network (answer options: yes/no, and if yes; specified categories of frequency).
3. Sociodemographic data included items on age and gender, patient self-report on current living and family situation and occupational status – all specially designed for the Network (answer options: specified categories of frequency).
4. All participants were diagnosed according to the DSM-5 [APA (38)] using the M.I.N.I. International Neuropsychiatric Interview [MINI (39)] for mental health disorders and the Structured Clinical Interview for DSM-5 Personality Disorders [SCID-5-PD; (40)]. Reliability of diagnostic interviews was not tested, but in both samples diagnostic assessments were performed by experienced clinicians with relevant training. The TS was assessed by clinical psychologists, psychiatrists, and resident doctors in psychiatry training. One participant was assessed by an experienced psychiatric nurse who had attended the preparatory workshops. In CS all diagnostic assessments are performed in a multidisciplinary team and concluded by a specialist in psychiatry/clinical psychology. In this study PDs are presented as diagnostic categories and PD severity is indicated by the number of fulfilled PD criteria and the number of fulfilled PDs.
5. Childhood trauma questionnaire [CTQ (41)] – a 28-item self-report questionnaire (1–5 scale) designed to assess the presence and severity of five types of negative childhood experiences: (1) emotional neglect (e.g., “I felt loved”), emotional abuse (e.g., “People in my family said hurtful or insulting things to me”), (3) physical neglect (e.g., “I did not have enough to eat”), (4) physical abuse (e.g., “I was punished with a belt, a board, a cord, or some other hard object”), and sexual abuse (e.g., “Someone tried to make me do sexual things or watch sexual things”).
6. Patient Health Questionnaire, Depression (PHQ-9) assesses depressive symptoms by nine items [0–3 response scale; (42, 43)]. In line with other population studies (44, 45), scores ≥10 indicate clinically relevant depressive states.
7. Generalized Anxiety Disorder-7 (GAD-7) assesses anxiety symptoms by seven items [0–3 response scale; (46)]. Scores ≥10 indicate a possible anxiety disorder, but scores ≥8 are also reported cut-offs (47).
8. PTSD Checklist for DSM-5 [PTSD-CL-5, (48)], is a 20-item self-report with 4 subscales: Intrusion (five items), Avoidance (two items), Negative alterations in cognitions and mood (seven items), and Alterations in arousal and reactivity (six items). The PTSD-CL-5 uses a 5-point Likert scale, symptoms during the past month, options ranging from “not at all” (0) to “extremely” (4). A score of 33 or higher is considered to indicate a possible diagnosis of PTSD according to the criteria of the DSM-5.
9. AUDIT (Alcohol Use Disorders Identification Test, WHO 2001) and DUDIT [Drug Use Disorders Identification Test; WHO 2001; (49)]. The study used the first 3 screening questions to identify magnitude of use and minimize the amount of questions asked in total. The sumscore of the three items is reported.
10. Levels of Personality Functioning–Brief Form [LPFS-BF 2.0 (50)] is a 12-item self-report measure of the DSM–5 Level of Personality Functioning Scale (Alternative Model of Personality Disorders, 5th edition of the Diagnostic and Statistical Manual of Mental Disorder), rated on a 0–3 response scale (“Very false or Often False” to “Very true or often True”). In this study, we present the mean sum-score of LPFS-BF. In a German study, population norms (T 50) were at score 15 (51), and a recent Danish study recommended that clinical dysfunction was indicated with increasing severity at scores >14 (52). LPFS-BF scores >14 thus indicate the severity of personality disorder.
11. The Global Functioning Scale [GFS; (53)] was used in both samples and is a revision based on the Global Assessment of Functioning Scale [GAF; APA (54)], and was rated by clinicians with score range 1–100, representing symptom severity and social impairment, reporting the more severe of the two. In a Norwegian study (55), reliability of the GAF was acceptable (generalizability coefficients relative decisions: 0.84 and absolute decisions: 0.82). Conventional interpretations of severity indicated by GAF are also applicable for GFS: Severe impairment indicated by scores = <50.
12. EuroQuol (EQ-5D-3L) is patient-reported health-related quality of life (56) with five specific items and a visual analogue scale (VAS), ranging health state from worst to best possible (scores 0–100). Mean VAS scores (burden of disease) in general population studies range 80–89 (57, 58).

### 2.6. More detailed investigation of TS

1. Lifetime parasuicide count [LPC (59)] is a specific enquiry on SH and SA by interview. Items include exploration of self-harm behaviors first time, most severe incident, last incident, age at the time of the incidents, suicidal intention, and resulting need for different health services.
2. Additional enquiry designed for the Extreme Challenges project:

a. SH/SA by patient report (Supplementary Table 3),

i. Expanded answer options on SH/SA frequency.
ii. Need for medical treatment, serious life threat, lasting physical sequelae, long-term injury (answer options, never, seldom, often)
iii. SH/SA incidents last 24 months (answer option yes/no)

b. Hospital treatment (patient and clinician report):

i. Hospital admissions due to self-harm? (Answer option yes/no)
ii. Age first hospital admission due to self-harm (specified categories)
iii. Current admission – duration (weeks)
iv. Number of admissions last 5 years
v. Duration of longest admission (weeks).

c. Language development: “At which age did you start to talk?” (answer options: normal: approx. 2 years; late: after 2.5 years; do not know).

3. Additional enquiry by clinician-report designed for the Extreme Challenges project

a. Prevailing physical health problems due to SH (answer option: yes/no)
b. Prevailing physical health problems of other cause (answer option: yes/no)
c. Other current physical disorders (open space for specification)

4. Additional diagnostic screening instruments.

1. Hayes Ability screening index, HASI (60) is a validated screening tool for intellectual disabilities and consists of six subtests of attention, memory, problem-solving, and language. HASI is designed to be completed in about 10 min and is applicable across age groups (age 13–adults), and settings (medical, psychiatric, and educational). The test has shown good validity with a cut-off <85 identifying 75% with borderline intellectual disability (61).
2. Ritvo Autism Asperger Diagnostic Scale-Revised, RAADS-R (62) is a self-report questionnaire based on the ICD-10 and DSM-5 diagnostic criteria used to assess the presence and severity of ASD (63). It consists of 80 items covering areas of social interaction, communication, and repetitive behaviors, with cut-off score > 65 (further investigation indicated), though by some recommended cut-off score > 72 (64). It is designed for individuals aged 18 years and older. If positive screening, further diagnostic evaluation was based on recommended guidelines from Oslo University Hospital.
3. DES-B: Brief Dissociative Experience Scale – Modified (65) is a patient self-report screening for dissociative disorder based on 8 items scored 1–5 (not at all, once or twice, nearly every day, about daily, more than daily. An example of the items is “People, objects, or the world around me seem strange or unreal.” In addition to the brief screening module within the M.I.N.I., DES-B was administered as an interview performed by clinicians. Systematic diagnostic evaluation was advised if the mean sum-score was >4, or on suspicion of dissociation raised by the screening interview.
4. If positive screening for ADHD based on the module within M.I.N.I, further evaluation was based on the DIVA 2.0 Diagnostic Interview for ADHD in adults (66).

### 2.7. Ethics statement

Participation required informed, written consent from participants in the inpatient target sample (project Extreme Challenges). Procedures for this data collection were approved by the Norwegian Regional Ethics Committee (REK; 2018/1124/REK Sør-Øst). The quality register of the Network for Personality Disorders is based on anonymous clinical data, and formal approvals from the Norwegian State Data Inspectorate and Medical Research and Ethics committee are not required. Written informed patient consent to contribute to the register is required. In both the inpatient target sample and the outpatient comparison sample, data collection procedures were approved by the local Data Protection Officer at each contributing hospital/treatment unit. Security procedures for the quality register are approved by the data protection officer at the responsible centre (Section for Personality Psychiatry and Specialized Treatments, Oslo University Hospital). The project Extreme Challenges has preregistration in Clinical Trials (NCT03768674).

### 2.8. Statistics

Statistical analyses were performed using software IBM SPSS version 28. Descriptive data are presented for continuous variables with means, standard deviations (SD), max-min values and median values, and categorical variables with valid per cent (%). Testing of differences between the inpatient target sample (TS) and the outpatient comparison sample (CS) were analyzed using Pearson Chi-Square test for categorical variables, descriptive continuous variables on work/study activity independent samples T-test and for the main analyses, comparing 1) mental health status, and 2) social functioning and quality of life, in CS and TS continuous variables were included in two separate one-way multivariate analyses of variance (MANOVA). Analyses 1 and 2 investigated each altogether eight dependent variables, variables with intercorrelation >0.5 were not included. Specified fixed effects from the MANOVAs are provided in Supplementary Tables 4, 5. For these continuous variables effect sizes are presented to further illustrate the magnitude of the differences - indicated by partial eta squared (ηp 2), where 0.01 is considered a small effect size, 0.06 moderate and 0.14 large. Taking the multiple comparisons of this study into account we have indicated non significance for all *p*-values >0.01. A strict Bonferroni correction would in our case entail a significance level of *p* < 0.001. We have chosen to present exact *p*-estimates for values between 0.01–0.001.

## 3. Results

### 3.1. Background factors

#### 3.1.1. Sociodemographic

Most individuals in TS were female (95%) and young adults (Age: *M* = 29, SD 8) and compared to CS, the distribution of age, and gender did not differ significantly (*p* > 0.01, Table 1). A majority of TS (63%) lived alone, versus 38% in CS (*p* = 0.05).

**Table 1:** Sociodemographic and background factors.

	TS		CS		Difference
	%	Mean (SD)	%	Mean (SD)	*p*
Female gender	95		79		ns
Age		29 (8)		30 (9)	ns
*Current living situation*					
Living alone	63		38		ns
Alone with child	5		6		
With parents	8		12		
With partner	13		28		
*Childhood trauma*					
Emotional abuse	52		43		ns
Physical abuse	21		14		ns
Sexual abuse	52		33		0.01
Emotional neglect	50		51		ns
Physical neglect	31		30		ns
*Education/work*					
Education after obligatory (years)		3.6 (3)		4.2 (3)	ns
<50% work/study last 6 months	87		48		0.003
*Health services*					
Former psych. outpatient treatment	100		83		0.004
Former psych. hospital admissions	100		31		<0.001

The table demonstrates current and background status among patients in the target sample (TS) and outpatients in the comparison sample (CS). Non-significance is indicated by ns (*p* > 0.01).

Childhood history of trauma was reported in both TS and CS, but compared to CS, data suggested a more frequent occurrence of sexual abuse in TS (*p* = 0.01).

#### 3.1.2. Hospital admissions

In TS, 79% reported more than 10 former admissions to a psychiatric hospital (ever), and 46% reported their first admission before age 18 years. Psychiatric hospital admissions were usually due to SH in 58%, always in 10%. Altogether 31% reported their first admission due to SH before the age of 18 years. Twenty-five percent reported their longest admission to be >6 months, in 22% the longest admission was before age 18 years. The first admission was due to SH in 45% (partly due to self-harm: 48%). By clinician report (clinical records) the longest admission in TS had lasted a median of 56 weeks (*M* = 89 weeks, SD 85). Median number of admissions last 5 years was 25 (*M* = 31, SD 25). Median duration of the current, ongoing admission was 7 weeks (*M* = 31, SD 68). Compared to CS, individuals in TS had significantly more former inpatient treatment experience (*p* < 0.001, Table 1).

#### 3.1.3. Self-harming behaviors (SH) and suicide attempts (SA) – occurrence and magnitude

In TS 93% reported lifetime SH (SH ever), also during the last 24 months, 61% reported more than 100 SH episodes, and 39% had self-harmed weekly or more often during the past 6 months (Table 2). Compared to TS, lifetime SH was also common in CS (74%, *p* = 0.02), but lifetime and current (last 6 months) SH magnitudes were significantly higher in TS (TS: 82% > 50 times, CS: 32% > 50 times, *p* < 0.001, Table 2). During the last 6 months, SH occurred more than twice as often in TS (*p* < 0.001, Table 2). In TS 95% reported lifetime SA, 76% had a SA during the last 24 months, 88% reported more than two attempts and 63% reported more than 5 attempts (Table 2). Differences between CS and TS were highly significant, compared to CS, lifetime occurrence and frequencies of SA in TS were doubled (*p* < 0.001, Table 2). During the last 6 months, three out of four TS participants reported SA during the last 6 months, compared to about 1 of 9 in CS (Table 2). In both samples, equivalent majorities in TS and CS reported first onset of SH and SA before the age of 18 (SH: 80–81%, SA: 58–63%, *p* > 0.05, Table 2).

**Table 2:** Self-harm and suicide attempts – occurrence and magnitude.

		Occurrence		Frequency	
		%	*p*_comparison_	%	*p*_comparison_
Self-harm					
Former acts ever	TS	93	0.02	>50 times: 82	<0.001
	CS	74		>50 times: 32	
First time age < 18	TS	80	ns		
	CS	81			
Last 6 months	TS	86	<0.001	> = weekly: 61	<0.001
	CS	41		> = weekly: 32	
Last 24 months	TS	93			
Suicide attempts					
Former acts ever	TS	95	<0.001	>twice: 88	<0.001
	CS	45		>twice: 44	
First time	TS	Age < 18: 58	ns		
	CS	Age < 18: 63			
Last 6 months	TS	74	<0.001		
	CS	12			
Last 24 months	TS	76			

The table demonstrates self-harm behaviors and suicide attempts reported by inpatients in the target sample (TS) and outpatients in the comparison sample (CS). Non-significant differences are indicated by ns (*p* > 0.01). In addition, reports on SH/SA last 24 months are given for the TS.

#### 3.1.4. Consequences of SH/SA in the target sample

In TS, 78% reported that SH episodes often lead to emergency treatments, and for 56%, often hospital treatment (patient self-report, Table 3). For 81% of TS participants, SA episodes were reported to often lead to emergency treatments, and for 75%, often hospital treatment (patient self-report, Table 3). In TS 53% reported that SH often resulted in severe life threat, 65% that SA often resulted in severe life threat, 45% reported lasting physical sequelae due to SH acts, and 34% due to SA (patient-report). By clinician report, 29% were currently considered to have persisting severe physical health problems due to SH/SA.

**Table 3:** Consequences of self-harm and suicide attempts in the target sample.

	Emergency room	Medical hospital
	%	%
*SH*		
Often leading to	78	56
First time	9	6
Last time	63	40
Most severe	89	74
*SA*		
Often leading to	81	75

The table demonstrates service-related consequences of self-harm (SH) and suicide attempts (SA) reported by inpatients in the target sample.

#### 3.1.5. Development of SH/SA in the target sample

In TS, all participants reported their last SH episode after the age of 18 and 88% indicated their most severe SH behaviors after the age of 18. The intention to die increased from the first SH episode (23%) to the last episode (58%). 83% reported a suicidal intention in the most severe SH episode.

#### 3.1.6. Other health problems among participants in the target sample

In TS current physical illness irrespective of SH/SA was reported in 63%. Type of illness was specified for 22 cases, among which 36% (*N* = 8) had more than one physical illness (mean number 1.6). Illnesses were specified within the following categories: Neurological 32% (*N* = 7), Immunological 23% (*N* = 5), Gastrological 23% (*N* = 5), Cardiovascular 14% (*N* = 3), Gynaecological 9% (*N* = 2), Muscle-Skeletal 32% (*N* = 7), and Other 32% (*N* = 7). In TS altogether 17% were reported to have current persisting severe health problems of other cause than SH/SA (clinician report).

### 3.2. Mental health disorder status

**Figure 1:**
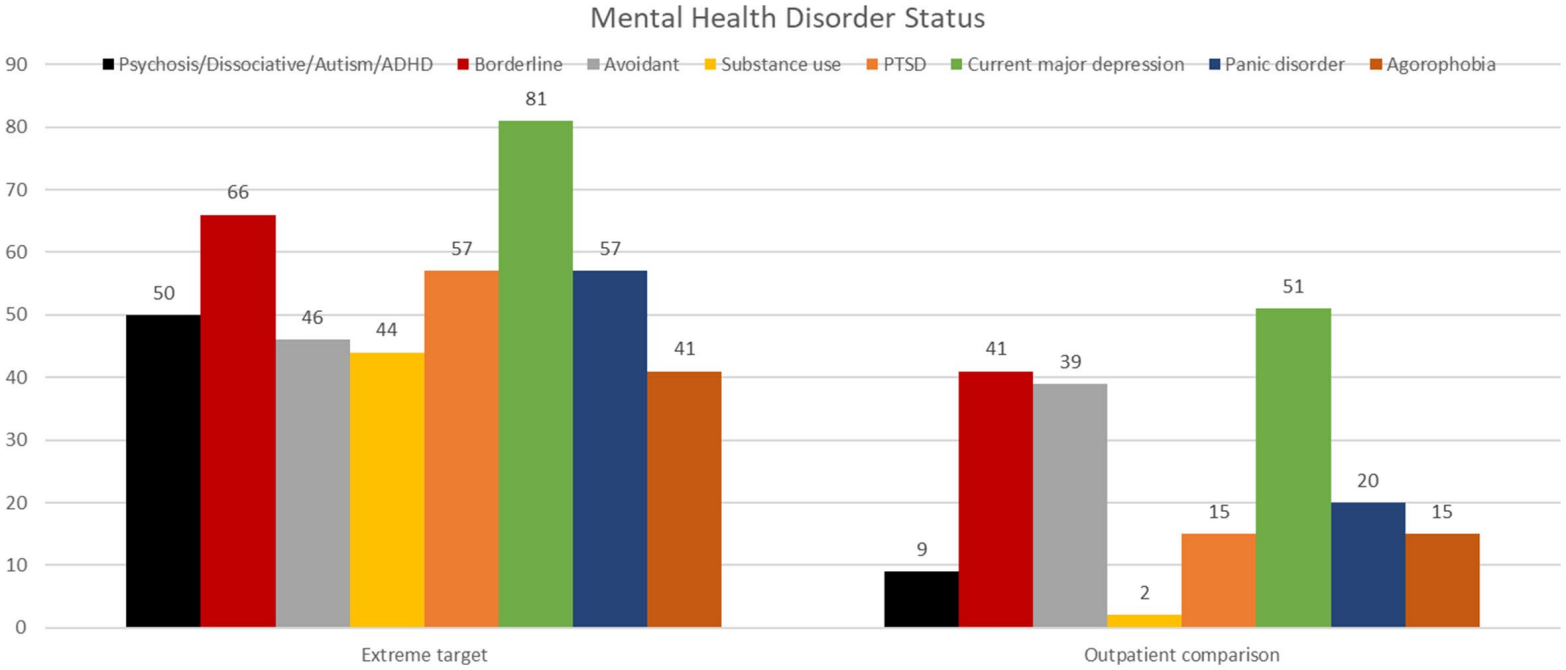
Demonstrates mental health disorders with frequencies >40% in the target sample, and corresponding frequencies in the comparison sample.

Table 4 demonstrates the distribution of mental health disorders as assessed by clinical interview and the symptom burden reported by participants and TS—CS comparison. Figure 1 demonstrates mental health disorder distribution in TS and CS.

**Table 4:** Mental health disorder status.

		TS		CS		Difference	
		%	Mean (SD)	%	Mean (SD)	*p*	*ηp 2*
Comorbidity and PD severity	No PD	9		23		ns	
	No other mental health disorder	0		36		<0.001	
	Number of other mental disorders		4.9 (2.4)		1.3 (1.4)	<0.001	0.39
	Number of SCID 5 PD criteria		14.6 (7.0)		11.9 (6.1)	ns	0.01
	LPFS-BF self-report		17.4 (7.3)		19 (6.0)	ns	0.001
Mood	Bipolar 1	8		2		ns	
	Bipolar 2	8		3		ns	
	Dysthymia	24		8		0.005	
	Current major depression	81		51		<0.001	
	PHQ-9 self-report		22.5 (4)		18.4 (5.1)	<0.001	0.07
Trauma	PTSD	57		15		<0.001	
	PTSD CL self-report		49.5 (20)		27.6 (23)	<0.001	0.11
Substance use	Alcohol misuse & dependency	41		8		<0.001	
	AUDIT screening sum-score		4.7 (3)		4.4 (2)	ns	0.001
	Substance use	44		2			
	DUDIT screening sum-score		2.1 (3)		0.5 (1)	<0.001	0.07
Anxiety	OCD	24		4		<0.001	
	Panic disorder	57		20		<0.001	
	Agoraphobia	41		15		<0.001	
	Social Phobia	38		25		ns	
	GAD	19		16		ns	
	GAD-7 self-report		15.3 (4.6)		13.6 (4.4)	ns	0.02
Somatic	Somatoform disorder	5		3		ns	
Eating	Eating disorder	19		9		ns	
Activity and concentration	ADHD	14		8		ns	
Psychosis and dissociation	Psychosis	14		0.5		<0.001	
	Dissociative disorder	11		0.5		0.002	
Developmental	Learning abilities - HASI total	35	86.5 (8)				
	Autism spectrum	11		0		<0.001	
	RAADS-R total self-report	48	84.5 (47)				
Personality disorder	Schizoid	0		1		ns	
	Schizotypal	3		0		ns	
	Paranoid	14		10		ns	
	Antisocial	3		2		ns	
	Narcissistic	0		0.3		ns	
	Borderline	66		41		0.007	
	Histrionic	0		0		ns	
	Avoidant	46		39		ns	
	Dependent	9		7		ns	
	Obsessive-Compulsive	14		6		ns	

The table demonstrates personality disorder (PD) and other mental health disorder status for inpatients in the target sample (TS) and outpatients in the comparison sample (CS). Significant differences between samples are indicated in the Table with *p* (Pearson Chi-Square) for categorical variables and continuous variables included in a multivariate analysis of variance with calculated effect sizes with Partial Eta Squared (*ηp 2*), non-significance is indicated by ns (*p* > 0.01). The table also demonstrates scores on autism screening (RAADS-R total) and cognitive, learning disability (HASI) in TS.

The overall mental health disorder status in TS was characterized by comorbidity across different categories of mental health disorders, significantly higher than CS (total number of mental health disorders other than PD: TS: 4.9, SD 2.4, CS:1.3, SD 1.4, *p* < 0.001, large effect size: 0.39). In TS most participants qualified for PD (91%) and in CS, PDs were also the largest diagnostic group (77%, Table 4). Severity of PD as indicated by the total number of PD criteria across categories (structured diagnostic evaluation by clinicians) and by patient self-report of levels of personality functioning (LPFS-BF) indicated impaired personality functioning in both groups with nonsignificant TS-CS differences and small effect sizes (*p* > 0.01, Table 4). The total number of comorbid PDs was not significantly higher in TS as compared to CS (TS: *M* = 1.6, SD 1.0, CS: *M* = 1.1, SD 0.9, *p* > 0.01).

In TS major depression was the most frequent disorder (81%) among mental health disorders other than PD. Clinician evaluation by structured interview was supported by patient self-report of depressive symptoms (PHQ-9: *M* = 22.5, SD 4). Although symptom levels were also high in CS (51%, PHQ-9 *M* = 18.4, SD 5.1), symptom levels were significantly higher in TS as compared to CS (*p* < 0.001, moderate effect size: 0.07, Table 4).

In TS PTSD was diagnosed for 57% of the participants (clinician evaluation) and represented together with panic disorder, the second most common disorder among mental health disorders other than PD. Compared to CS (15%), it was significantly more frequent in TS (*p* < 0.001, Table 4). Participant-rated severity of trauma-symptoms (PTSD-CL) was above cut-off 33 in 76% of TS and 45% of CS (PTSD-CL TS: *M* = 49,5, SD 20, CS: *M* = 27.6, SD 23, *p* < 0.001, large effect size: 0.11).

In TS 41–44% qualified for alcohol or substance use disorder, respectively (Table 4), frequencies were significantly higher in CS than TS (2–8%) (Table 4). Comparing CS and TS self-reports (AUDIT and DUDIT), only use of other substances than alcohol differed significantly (*p* < 0.001, moderate effect size: 0.07) from CS (Table 4).

In TS, panic disorder was the most frequent of anxiety disorders (57%). Compared to CS, panic disorder (TS 57% CS: 20%), OCD (TS: 24%, CS: 4%), and agoraphobia (TS: 41%, CS: 15%) were all significantly more frequently diagnosed in TS (*p* < 0.001, clinician administered structured interview). Anxiety diagnoses were supported by patient self-report levels of anxiety symptoms. The intensity of symptoms in TS and CS were not significantly different and the effect size was small (GAD-7 TS: *M* = 15.3, SD 4.6, CS *M* = 13.6, SD 4.4, small effect size: 0.02, *p* > 0.01, Table 4).

Comparing CS and TS, significant differences were found (clinician evaluation) for psychoses (TS: 14%, CS: 0.5%, *p* > 0.001), dissociative disorder (TS: 11%, CS: 0.5%, *p* = 0.002), and ASD (TS: 11%, CS: 0%, *p* < 0.001), but not for ADHD (TS: 14%, CS: 8%, *p* > 0.01) (Table 4). Pooling these four disorders, altogether 50% of TS had at least one of these disorders versus 1% in CS (TS-CS difference: *p* < 0.001).

In TS screening of intellectual disability with HASI indicated a need for further investigation in 35%. For 10%, patient self-report indicated late language development in childhood. Five participants in TS lacked information from diagnostic interviews but had patient self-report.

In TS, ASD screening with RAADS-R indicated a need for further investigation for a larger proportion than those who had received a diagnosis at the point of the investigation (RAADS-R cut-off 65: 55%/cut-off 72: 48%, *M* = 84.5, SD 47, Table 4).

Comparing TS and CS, differences in frequencies of most specific PD categories were not significant (*p* > 0.01, Table 4). Data suggested that BPD was more frequent in TS than CS (*p* = 0.007).

### 3.3. Psychosocial functioning and health-related quality of life

Table 1 demonstrates % with <50% work/study activity last 6 months. Mean number of months in work or study was low in both TS (*M* = 0.37, SD 1.1) and CS (*M* = 2.50, SD 2.8). Comparing TS and CS, rates were significantly lower in TS (*p* = 0.003). In TS a total of 82% had disability benefits or work allowance, compared to 38% in CS (*p* < 0.001).

Table 5 demonstrates social functioning and health related quality of life. Based on clinicians’ and participants’ global rating of functioning and health-related quality of life, respectively, participants in both samples indicated impairments over clinical cut-off. Comparing TS and CS, differences between the samples were large for psychosocial functioning (GFS: TS: *M* = 39, SD 8, CS: *M* = 52, SD 6, *p* < 0.001, large effect size: 0.28), significant, but less for health-related quality of life (EQ 5D VAS: TS: *M* = 37, SD 22, CS: *M* = 49, SD 19, *p* < 0.006, small effect size: 0.02). Differences indicated poorer functioning and quality of life in TS. The specified elements of health-related quality of life differed only on the item on personal management (*p* < 0.001, effect size moderate: 0.05, Table 5).

**Table 5:** Social functioning and health-related quality of life.

	Target inpatient		Comparison outpatient		Difference	*ηp 2*
	%	Mean (SD)	%	Mean (SD)	*p*	
	Score < 50		Score < 50			
GFS – clinician rated	86	39 (8)	69	52 (6)	<0.001	0.28
EQ 5D VAS – patient self-report	73	37 (22)	46	49 (19)	0.006	0.02
EQ 5D 3 L items:	*Score = > 2*		*Score = > 2*			
1: Movement problems	32		26		ns	0.01
2: Personal management, hygiene/clothing	51		22		<0.001	0.05
3: Social activity, work/study/housework/leisure	88		81		ns	0.007
4: Pain problems	66		68		ns	0.001
5: Anxiety/Depressive symptoms	100		98		ns	0.02

The table demonstrates clinician-rated global functioning (GFS), and aspects of health-related quality of life (EQ 5D 3 L) reported by inpatients in the target sample (TS) and outpatients in the comparison sample (CS). Significant differences between samples are indicated in the table with *p*. Analyses of categorical variables were based on Pearson Chi-Square. Continuous variables were included in a multivariate analysis of variance and calculated effect sizes indicated by Partial Eta Squared (*ηp 2*). Non-significance is indicated by ns *(p* > 0.01).

## 4. Discussion

This study recruited individuals characterized by extensive psychiatric hospitalization resulting from severe or repetitive self-harming behaviors. There are few studies of such extreme situations and systematic prevalence estimates are lacking. Nonetheless, participants with high-risk behaviors, under repeated suicidal threat and long-term or frequent hospital treatment present a considerable challenge to health services and the situations are highly burdensome for the individual, their families and network. As expected, the target sample (TS) in this study contrasted the comparison sample (CS) with respect to the extent of self-harming behaviors and experiences of hospitalization. The aim of the present research was to investigate aspects of mental health disorder, psychosocial functioning, and quality of life and characterize differences between these two populations – the first representing a severe and high-risk inpatient situation, the second representing patients found eligible for further psychotherapeutic treatment.

### 4.1. Main findings

Our main findings are the following:

1. Current self-harming behaviors were more extensive in TS than CS, but age of onset did not differ. In TS participants reported a development where self-harming behaviors had increased in severity and suicidal intention.
2. PD was diagnosed in the majority of TS and was the most frequent disorder in both samples. Compared to CS, personality functioning was not more impaired. In TS the most frequent comorbid conditions included major depression, PTSD, substance use disorders, and panic disorder. Compared to CS, mental health disorder status was significantly more complex and severe with higher comorbidity of other mental health disorders. Significant differences between CS and TS included higher frequencies of psychoses, dissociative disorder, and developmental disorders in TS. Noteworthy indications of possible learning disabilities were found in TS.
3. Impairments of psychosocial functioning and health-related quality of life were evident in both samples, but more so in TS. Large proportions were completely outside the work force, and occupational impairment was greater in TS than CS.

#### 4.1.1. Development of severe self-harm

Prospective studies specifically focusing on the severe segment of self-harming individuals are largely lacking; little is known about their longitudinal development of self-harming behaviors from childhood to adult age. However, high repetition self-harm has been linked to long-standing psychosocial vulnerabilities (8), and children exposed to abuse or neglect have increased risk for suicidal ideation and self-harm in middle childhood, with unique developmental pathways for these behaviors (67). In our study, more than half of the participants in TS reported childhood sexual abuse.

In the present study we found that self-harming behaviors and suicidal intention increased over time in TS. These individuals were characterized by considerable exacerbation over time. Other research has associated repeated self-harm periods and high volumes of self-harming behaviors with generally increased mortality and completed suicides (16, 68). The present study thus targets individuals who represent a high-risk cohort.

The escalated development is sharply contrasted to the more general tendency where self-harm tends to decline in adult populations (69, 70). Across populations, studies indicate that the majority who self-harm do not develop chronic or habitual self-harm (71). For BPD in particular, longitudinal studies have demonstrated that self-harming behaviors are among features most likely to remit over time (72, 73). Such remission is also frequently reported as positive outcomes in effective treatments for BPD (74). However, in a twenty-year, longitudinal study from adolescence to adulthood, Cohen et al. (75) not only pointed to a majority with remission of BPD traits, but also indicated that 20% of the adolescents had persisting PD problems in adulthood.

Based on our current investigation it is conceivable that the developments described in the present sample of severely self-harming individuals mainly reflect a process of considerable mental health burden and deepening despair during the transition from adolescence into adulthood. Self-harm used as affect regulating coping mechanisms might over time lose its effectiveness, and the behaviors thus become more frequent and/or severe. Underlying problems may be difficult to access, stay unresolved or worsen as life evolves. Indeed, severe self-harm and suicide attempts have also in older populations been associated with the accumulation of feelings of loneliness, loss of control, and increasing physical and mental health challenges (76). Further investigation of the different motivations and mechanisms present among individuals with severe self-harming behaviors and how they are met within health services are highly relevant, though outside the scope of the present study which was limited to an investigation of mental health status.

#### 4.1.2. Complexity of psychopathology

The present study revealed a dominance of PD, BPD in particular, coupled with considerable comorbidity of other mental disorders (mood, anxiety, PTSD and substance use) and noteworthy concentration of severe disorder (psychosis, dissociative disorder, ASD) in TS. Although small numbers, the latter were significantly less frequent in CS. The complexity is further underlined by the high rates of possible intellectual disability indicated in TS, and reports of co-occurring somatic complaints. Although research and treatment recommendations are often limited to specific mental disorders, associations between extensive self-harm/suicide and severe psychopathology have been emphasized in previous studies demonstrating a broader range of physical and mental illness comorbidity, trauma, and interpersonal problems (77). Our results and related research indicate that a one-sided disorder specific focus may facilitate an underscoring of the total situation and thus also an overestimation of the person’s capacities. A negative or lack of development due to inadequate or poorly adapted treatment could also be part of this picture.

The high occurrence of BPD was expected. However, a perhaps more surprising result was that TS also included a large proportion of individuals with avoidant PD features, thus depicting participants with considerable problems of restriction and introversion. Within the field of PD, self-harm and suicide risk is a main focus when approaching individuals with BPD. However, other PDs and correspondingly adjusted therapies are to a less extent developed and investigated, even though such challenges are increasingly acknowledged (78). Several studies of avoidant PD emphasize the considerable psychosocial burden of this disorder (79, 80). When considering the current target population, the impact of avoidant PD alone or in concert with BPD should not be underestimated and may in part explain the seemingly lack of treatment response in the years preceding the current target situation.

Major depression was the most common comorbidity and anxiety disorders were also common. Such high burden of symptoms qualifying for comorbid diagnoses is frequently reported in clinical samples with PD (36, 81, 82) and may be driven by the personality pathology. Nonetheless, compared to CS, frequencies in TS were higher. In a 24-year follow-up of poorly functioning inpatients with PD, rates of depression were generally high – on admission to hospital 87% of BPD patients and 76% of other PDs had major depression. Among the inpatients with BPD and major depression, two-year remission was found for 93%, but also recurrence for 90%. Among patients without major depression at intake, 86% experienced new occurrence (83). The high rates of depression among these inpatients are comparable to TS, and the levels of global functioning indicated equivalent and severe impairment (GAF 39). In a quite recent study comparing adolescent and adult patients with BPD, comorbidity of mood and anxiety disorders was higher among adults, thus suggesting a process of increasing burden over time with a pattern of more complex comorbidity becoming apparent in adulthood (84).

Our participants in the target group showed a high degree of PTSD. Although childhood trauma was frequent in both groups, our data suggested an overrepresentation of sexual abuse in the target group. Levels of diagnosed PTSD by clinician administered interview and evaluation were generally lower than indicated by patient self-report. However, by both assessment methods, trauma-related problems were more frequent in the target sample. It may thus be concluded that PTSD was a significant factor for a majority of this sample. Persons with PTSD and complex PTSD are known to be more at risk of self-harm (85, 86). It is also suggested that shame can be an important emotion in self-harm (87), and in connection to childhood sexual abuse, bodily shame has been associated with self-harming behavior (88).

A common comorbidity was substance abuse disorder. By clinical assessment nearly half of the TS participants qualified. In our study, rates of alcohol dependency and substance use were significantly higher in TS than CS. Substance use is normally excluded from the definition of self-harm, but is a frequent co-occurring diagnosis (89, 90). Globally, substance use and dependency is diagnosed in around 25% of suicide completers (91) and is significant among youths who have traversed from suicidal ideation to attempts (92). This highlights the importance of treatment and interventions for the individuals concerned. Among people with substance use disorders there have been few guidelines on how to manage suicide risk or self-harm and little evidence for effective interventions (93). Seeing the high rate of substance use in our severely self-harming participants, this should be amended through targeted research and clinical implementation.

ASD was signaled by screening with RAADS-R in near half of TS. A high RAADS-R score does not necessarily equal a diagnosis of ASD. In possible support of recently reported validity concerns (94), the percentage of RAADS-R screening over cut-off in our study, was considerably higher than the full diagnostic evaluation. High screening scores are still conspicuous and worthy of further, more detailed exploration of clinically notable problem areas. Previous findings indicate that ASD may be overlooked or misunderstood in general mental health services (95, 96). Thus, some of the participants with high scores on the RAADS-R may have an undiagnosed ASD. Considering the core aspect of ASD, social cognition, in a dimensional perspective, impairments also below threshold for diagnosis may have clinical relevance across mental health disorders.

ASD and BPD share some features, including social and emotion regulation difficulties, similarities which have spurred research on the potential overlap of these diagnoses, though without clear results (97). Nonetheless, studies have reported enhanced autistic trait levels among people with BPD (98). The association between ASD and self-harm as a distress response is yet to be established (99). Nonetheless, individuals with co-occurring BPD and ASD (63) are known to be at risk for more suicide attempts and lower general functioning (100). Moreover, studies comparing social cognition capacities across persons with PD, schizophrenia, and normal controls, conclude that detectably poorer capacity is associated with schizophrenia and increasing comorbidity of PD, the latter more so than BPD alone (101, 102). Closely related to social cognition, a study comparing avoidant PD and BPD indicated poorer emotional awareness among individuals with avoidant PD (103). Impaired social cognition and autistic features have consequences for the choice of treatment intervention and if undetected, may complicate the treatment alliance. Further illustrating the possible interplay of patients’ social capacity and psychological treatments, in a small study of BPD patients in specialized treatment, more extensively impaired social cognition was associated with more childhood trauma, comorbid avoidant PD traits and/or PTSD, more extensive prior treatment experience, and poorer outcome with greater treatment irregularity (104).

The cognitive screening HASI showed that a noteworthy 35% of the participants qualified for continued assessment of possible intellectual disabilities. Among people with significant intellectual disabilities, severe self-harm is indeed seen to be more persistent, with poorer expressive communication as one of the more prominent features (105). Difficulties expressing oneself may lead to considerable mental and emotional stress and has the potential to push vulnerable persons into expressing themselves or releasing tension through self-harming behavior. The present study is however, limited by only including a screening test of such disability.

Psychosis was also a notable aspect of the target group. A recent study indicated that self-harm and suicidal attempts prior to psychotic episodes is associated with a more severe course of psychotic illness. There, self-harming individuals who developed psychotic episodes had more severe depressive and anxiety symptoms than individuals with psychosis, but no self-harm or suicide attempts (106). Furthermore, studies indicate that individuals with combined PD and psychosis have significantly higher rates of attempted or completed suicide compared with those without comorbid PD (107). Taken together, the connection between self-harm and severe medical conditions and psychiatric disorder seems striking, and self-harm can as such serve as a red flag for comorbid or prospective severe mental disorder.

In contrast to the present findings and studies reporting substantial differences in terms of consequences and comorbidities between BPD and non-BPD persons with SH (3), SH seems in clinical practise, often to be connected to the diagnosis of BPD. Our study of severe cases suggests that such an assumption may not provide a sufficient picture, and on the contrary, could limit the individual’s possibilities for treatment, more refined investigation, support, or rehabilitation. A consequence of such a possible “borderline trap” in the clinical environment may be that individuals continue to seek extensive emergency services due to unavailability of other options. Future research and not least, guidelines for self-harm need to capture the diagnostic heterogeneity and severity of overall mental health condition in order to tailor appropriate interventions and preventive measures.

Emotional disturbances and difficulties as well as different emotion-regulating strategies are central to many models of psychopathology (108, 109). In our target sample 91% of the participants qualified for PD, and the majority had additional symptom disorders. Diagnoses of BPD, PTSD, mood and anxiety disorders were all highly represented and clearly more frequent in this sample. All these conditions reflect problems effecting emotional states and regulation and their combination may indicate enhancement. The present results may also suggest difficulties identifying and regulating emotions as an underlying factor. Former studies have repeatedly shown emotional dysregulation as a driver for self-harm (110–112). Intrapersonal functions for self-harm are seen to be most common (66–81%, 113), with emotion regulation reported to account for 63–78%. Similar results for severely self-harming inpatients are shown in a Swedish study (114). Further, more detailed investigation of specific aspects of personality functioning across different conditions is warranted, and also scheduled within the Extreme Challenges project.

#### 4.1.3. Impairment of psychosocial functioning and quality of life

It is natural to assume that the total picture of severity of condition and complex comorbidity within multiple areas would affect every-day functioning and quality of life. Our study also provides some indication of poor physical health both related and unrelated to self-harm. The direction of causality may be uncertain or vary, but ill health is generally also known to be a negative prognostic factor for the severity of self-harm and suicide risk (15, 115, 116). In our study, a heavy global burden was indicated by poorer levels of psychosocial and occupational functioning, subjective experience of quality of life, and greater problems with personal management in the target group as compared to the outpatients. The levels of symptoms of anxiety and depression by patient report coincided with the interview-based diagnostic evaluation with significantly higher scores in the target group.

We do not know the trajectories of mental disorder development from adolescence to adulthood. However, it cannot be ruled out that the described weight of symptoms, poor general and social functioning, not least if combined with experiences of inadequate help, might have contributed to a spiraling of self-harm, possibly in an attempt to (temporarily) ease what is felt as an unbearable situation. Further studies adding perspective on the use of health services, collaborations, and qualitative exploration of personal experiences in the target group are scheduled in the Extreme Challenges project.

### 4.2. Strengths and limitations

The current study investigates an under-studied and poorly investigated severely self-harming population, in a national study with participants from all health regions of Norway. It is a naturalistic study where participants were recruited from real life inpatient situations and assessed by regular clinicians at the hospitals. Its clinical representatively is therefore high. It is also a strength of the study that the target group could be compared to a sizable and clinically representative outpatient sample of individuals admitted to regular personality disorder treatment within secondary mental health services in different parts of Norway, assessed with the same instruments/interviews and in the same time-period.

The target situation is severe, but not common, and the target sample is thus small (*N* = 42). However, the project recruited the stipulated frequency of patients per hospital (9) and the target sample hospitals’ geographic location reflected all health regions in Norway. Comparably, high-volume repeaters (>15 emergency help attendance within 4 years) made up 0.6% of the self-harm populations in a British study, but accounted for 10% of all emergency help self-harm attendances (68). Nonetheless, due to the limited sample size we have in this study presented descriptive data and compared two independent groups.

In both samples diagnoses were based on clinicians’ evaluation using systematic and structured assessment interviews. It is a limitation that the clinical situation did not enable reliability tests of diagnostic evaluations. However, clinicians in TS and CS received equivalent and systematic training on use of structured interviews and tests and principles of scoring. Also, diagnostic interviews were supplemented by patient self-report on symptoms of depression, anxiety, traumatization, and substance abuse. Both are reported in tables and text.

Limitations of RAADS-R as a screening test for ASD have recently been reported (94). Multiple measures are generally recommended for assessing ASD in clinical practice (117), as with other clinical assessments. In our study, additional assessment with specific instruments was advised when an ASD was suspected. Our study reports results from RAADS-R screening as well as results from extended, full diagnostic evaluation.

As the study has a cross-sectional design, it does not provide longitudinal data on the further development for persons engaging in severe self-harm after the inpatient treatment. The impacts of the current structured diagnostic assessment – more detailed than often provided during hospitalization – can therefore not be concluded.

## 5. Conclusion

The study reveals severe self-harm inpatients with complex psychopathology and highlights the importance of individualized and thorough assessment among patients displaying a development of increasingly severe and/or repetitive self-harm. The complex and overall massive pathology, both of psychiatric and physical ailments, clearly points to severe underlying pain, creating composite and both unique yet recognizably severe symptomatic pictures. These individuals are highly vulnerable, in need of thorough assessment, treatment and care. Preferably, our study will add knowledge and insight into this largely overlooked heterogeneous patient ‘group’, as well as open up a curiosity around them, which will serve health practitioners and legislators alike.

## Data availability statement

The datasets presented in this article are not readily available because the data used in this study are partly based on a quality register of the Norwegian Network for Personality Disorders and partly based on a multicenter data collection of inpatients. Due to restrictions regarding highly sensitive patient data, data are only available on specific request. Requests may be sent to the Privacy and Data protection Officer at Oslo University Hospital; personvern@ous-hf.no. Requests to access the datasets should be directed to personvern@ous-hf.no or corresponding author, EK e.h.kvarstein@medisin.uio.no, or TL, tuvalang@gmail.com.

## Author contributions

TL: Data curation, Formal analyses, Investigation, Project administration, Visualization, Writing - original draft, Writing - review & editing. GP - Data curation, Formal analyses, Investigation, Project administration, Resources, Software, Writing - review & editing. TB - Conceptualization, Resources, Writing - review & editing. TC - Conceptualization, Resources, Writing - review & editing. IE - Conceptualization, Writing - review & editing. OH - Conceptualization, Writing - review & editing. AK - Conceptualization, Writing - review & editing. EM - Conceptualization, Writing - review & editing. AN - Conceptualization, Resources, Writing - review & editing. RR - Conceptualization, Writing - review & editing. PR - Conceptualization, Resources, Writing - review & editing. KR - Conceptualization, Writing - review & editing. JS - Conceptualization, Resources, Writing - review & editing. TS - Conceptualization, Writing - review & editing. LS - Conceptualization, Writing - review & editing. TeT - Conceptualization, Resources, Writing - review & editing. MP - Conceptualization, Writing - review & editing. ToT - Conceptualization, Resources, Writing - review & editing. ØU - Conceptualization, Writing - review & editing. FW - Conceptualization, Writing - review & editing. EK - Conceptualization, Formal analyses, Funding acquisition, Investigation, Methodology, Project administration, Supervision, Visualization, Writing - original draft, Writing - review & editing.
