# Supplementary material for: Mental health disorders, functioning and health-related quality of life among extensively hospitalized patients due to severe self-harm – results from the Extreme Challenges project
Source: Front Psychiatry. 2023 Oct 18;14:1258025. doi: 10.3389/fpsyt.2023.1258025 (PMC10619742; doi:10.3389/fpsyt.2023.1258025)
Supplement: Supplementary file 1 [file Table_1.docx]

Supplementary Material

**Extreme Challenges: Mental health disorders, functioning and health-related quality of life among extensively hospitalized patients due to severe self-harm**

Tuva Langjord^*^, Geir Pedersen, Tone Bovim, Tore Buer Christensen, Ingeborg Ulltveit-Moe Eikenæs, Oddbjørn Hove, Arvid Nikolai Kildahl, Erlend Mork, Astrid Berge Norheim, Ruth Kari Ramleth, Petter Andreas Ringen, Kristin Lie Romm, Johan Siqveland, Thea Schønning, Line Stänicke, Terje Torgersen, Mona Pettersen, Tone Tveit, Øyvind Urnes, Fredrik Walby, Elfrida Hartveit Kvarstein

*** Correspondence:**
tuvalang@gmail.com

**Supplementary file 1a Inpatient target sample – recruitment of participating hospitals and patients**

| HEALTH REGIONS | Invited  Health Trust/Hospital | Accepted invitation | Preparatory clinician training course | Recruited patients | Number of patients | Patients per region |
| --- | --- | --- | --- | --- | --- | --- |
| South-eastern | *East* |  |  |  |  |  |
|  | 1 | Yes | Yes | Yes | 3 | 15 |
|  | 2 | Yes | Yes | Yes | 2 |  |
|  | 3 | Yes | Yes | Yes | 1 |  |
|  | 3 | Yes | Yes | No |  |  |
|  | 5 | Yes | Yes | No |  |  |
|  | 6 | No |  |  |  |  |
|  | 7 | No |  |  |  |  |
|  | *South* |  |  |  |  |  |
|  | 8 | Yes | Yes | Yes | 2 |  |
|  | 9 | Yes | Yes | Yes | 7 |  |
|  | 10 | Yes | Yes | No |  |  |
|  | 11 | No |  |  |  |  |
| Western | 12 | Yes | Yes | Yes | 3 | 7 |
|  | 13 | Yes | Yes | Yes | 1 |  |
|  | 14 | Yes | Yes | Yes | 3 |  |
|  | 15 | No |  |  |  |  |
|  | 16 | No |  |  |  |  |
| Mid | 17 | Yes | Yes | Yes | 9 | 13 |
|  | 18 | Yes | Yes | Yes | 4 |  |
|  | 19 | No |  |  |  |  |
| Northern | 20 | Yes | Yes | Yes | 2 | 7 |
|  | 21 | Yes | Yes | Yes | 5 |  |
|  | 22 | No |  |  |  |  |
|  | 23 | No |  |  |  |  |
| **SUM** | **23** | **15** | **15** | **12** | **42** | **42** |

**Supplementary file 1b Comparison outpatient comparison sample – Network for Personality disorder**

| HEALTH REGIONS | Units participating in data collection  to the Network for Personality Disorders quality register  period 2019-2020 | Number of Units | Number of patients | Patients per region |
| --- | --- | --- | --- | --- |
| South-eastern | *East* |  |  | 193 |
|  | 1 | 1 | 44 |  |
|  | 2 | 1 | 93 |  |
|  | 3 | 1 | 36 |  |
|  | 4 | 2 | 20 |  |
|  | *South* |  |  | 112 |
|  | 5 | 1 | 42 |  |
|  | 6 | 2 | 70 |  |
| Western | 7 | 2 | 54 | 83 |
|  | 8 | 2 | 29 |  |
| Mid | 9 | 1 | 1 | 1 |
| **SUM** |  | **13** | **389** |  |

**Supplementary file 2 ­– The total Extreme Challenges project assessment package**

| **MEASURING** | **INSTRUMENT** |
| --- | --- |
| Information on self-harm and other background factors | Self-harm acts – interviews and self-reports |
|  | Current sociodemographic status – self-report |
|  | Childhood Trauma Questionnaire (CTQ) |
| Mental health status assessed by clinician interview and supplemented by self-report | MINI interview mental health disorders |
|  | Patient Health Questionnaire Depression (PHQ-9) – self-report |
|  | General Anxiety Disorder-7 (GAD-7) - self report |
|  | PTSD Checklist for DSM-5 (Ptsd-CL-5) - self-report |
|  | Dissociative Experiences Scale - Brief form (DES-B) - administered as interview |
|  | Ritvo Autism Asperger Diagnostic Scale-Revised (RAADS-R) – self-report |
|  | Alcohol Use Disorders Identification Test (AUDIT) – self-report |
|  | Drug Use Disorders Identification Test (DUDIT) – self-report |
|  | SCID 5 PD interview personality disorders |
|  | Level of Personality Functioning Scale – Brief Form (LPFS-BF) – self-report |
| Cognitive abilities screening | The Hayes Ability Screening Index (HASI) - clinician administrated test |
| Social functioning and health-related quality of life | EuroQuoL (EQ-5D)- self report |
|  | Global Functioning Scale (GFS) – clinician rated |
| * | Work and Social Adjustment Scale (WSAS) – self-report |
| *Specific aspects of personality functioning | Severity Indices of Personality Problems (SIPP) – self-report |
|  | Toronto Alexithymia Scale (TAS-20) – self-report |
|  | Difficulties in Emotion Regulation Scale (DERS) – self-report |
|  | Supplementary clinician interview on dysregulated episodes (MBI-based) |
|  | Experiences in Close Relationships (125) – self-report |
|  | Modifed Overt Aggression scale (MOAS based) – self-report |
| *Health service utilization and collaborations | Health and welfare services past 6 months - interview |
|  | Previous treatment - self-report and clinician report |
|  | Cooperation within and between health care services - clinician and self-report |
| *Qualitative enquiry | Open spaces allowing patients and clinicians commentary on current situation |

Rows marked with * indicate assessments in the total package which were not focused in the present study, but are bases for other, separate studies within the Extreme Challenges project.

**Supplementary file Table 3 Patient-report on self-harm and suicide attempts**

|  | **Self-harm** | **Answer option TS** | **Answer option CS** |
| --- | --- | --- | --- |
| 1 | Have you ever on purpose harmed yourself? (eg cutting, burning, headbanging etc) | Yes/no | Yes/no |
|  | *If yes:* How many times have you self-harmed? | Up to 10 times, 10-50 times, 50-100 times, More than 100 times. | Only once, 2-5 times, 6-10 times, 11-50 times, More than 50 times. |
| 2 | *If yes:* How old were you the first time? | Before 13 years of age, 13-18 years, 19-29 years, in my thirties, in my forties, 50 or older. | Before 13 years of age, 13-18 years, 19-29 years, in my thirties, in my forties, 50 or older. |
| 3 | Has self-harm lead to medical treatment? | Never, Seldom, Often |  |
| 4 | Have you been admitted to medical/surgical hospital departments due to selfharm? | Never, Seldom, Often |  |
| 5 | Has self-harm lead to serious life threat? | Never, Seldom, Often |  |
| 6 | Has self-harm had lasting physical consequences? | Yes/no |  |
| 7 | Has self-harm lead to long-term physical injury? | Never, Seldom, Often |  |
| 8 | Have you purposely self-harmed the last six months? | Yes/no | Yes/no |
|  | *If yes:* How often have you self-harmed? | Daily, weekly, once a month or less | Daily, weekly, once a month or less |
| 9 | Have you purposely self-harmed last 24 months? | Yes/no |  |
|  | **Suicide attempts** | **Answer option** |  |
| 1 | Have you ever purposely tried to kill yourself? | Yes/no | Yes/no |
|  | *If yes:* How many times have attempted suicide? | Only once, twice, More than two times, More than five times | Only once, twice, More than two times. |
| 2 | *If yes:* How old were you the first time? | Before 13 years of age, 13-18 years, 19-29 years, in my thirties, in my forties, 50 or older. |  |
| 3 | *If more than twice*: Have suicide attempts lead to medical treatment? | Never, Seldom, Often |  |
| 4 | Have you been admitted to medical/surgical hospital departments due to suicide attempts? | Never, Seldom, Often |  |
| 5 | Have suicide attempts lead to serious life threat? | Never, Seldom, Often |  |
| 6 | Have suicide attempts had lasting physical consequences? | Yes/no |  |
| 7 | Has self-harm lead to long-term physical injury? | Never, Seldom, Often |  |
| 8 | Have you purposely attempted suicide the last six months? | Yes/no | Yes/no |
| 9 | Have you purposely attempted suicide the last 24 months? | Yes/no |  |

The items are developed in collaboration with the Norwegian Network for Personality Disorder, the research group Personality Psychiatry, University of Oslo, and the Extreme Challenges project group. The more detailed enquiry in items 3-7, item 9 on self-harm and on suicide attempts were specially developed for TS, but not a part of data registered in the quality registry from which CS was recruited. In TS the answer options for the number of self-harm incidents/suicide attempts was also expanded. English translation was by the last author of the present study.

**Supplementary file: Table 4 Mental disorders MANOVA**

| **Table 4a: Multivariate Tests^a^** | | | | | | | |
| --- | --- | --- | --- | --- | --- | --- | --- |
| Effect | | Value | F | Hypothesis df | Error df | Sig. | Partial Eta Squared |
| CS vs TS | Pillai's Trace | ,470 | 29,729^b^ | 8,000 | 268,000 | <,001 | ,470 |
|  | Wilks' Lambda | ,530 | 29,729^b^ | 8,000 | 268,000 | <,001 | ,470 |
|  | Hotelling's Trace | ,887 | 29,729^b^ | 8,000 | 268,000 | <,001 | ,470 |
|  | Roy's Largest Root | ,887 | 29,729^b^ | 8,000 | 268,000 | <,001 | ,470 |
| a. Design: Intercept + CS vs TS | | | | | | | |
| b. Exact statistic | | | | | | | |

| **Table 4 b Tests of Between-Subjects Effects** | | | | | | | |
| --- | --- | --- | --- | --- | --- | --- | --- |
| Source | Dependent Variable | Type III Sum of Squares | df | Mean Square | F | Sig. | Partial Eta Squared |
| CS vs TS | Number of symptom disorders | 358,514 | 1 | 358,514 | 172,152 | <,001 | ,385 |
|  | Number of SCID 5 PD criteria | 134,090 | 1 | 134,090 | 3,842 | ,051 | ,014 |
|  | PHQ-9 | 525,070 | 1 | 525,070 | 20,127 | <,001 | ,068 |
|  | GAD-7 | 85,146 | 1 | 85,146 | 4,416 | ,037 | ,016 |
|  | LPFS-BF | 16,616 | 1 | 16,616 | ,401 | ,527 | ,001 |
|  | PCL-5 sumscore | 17858,854 | 1 | 17858,854 | 35,101 | <,001 | ,113 |
|  | AUDITscreening sum-score | 1,458 | 1 | 1,458 | ,257 | ,612 | ,001 |
|  | DUDITscreening sum-score | 62,806 | 1 | 62,806 | 21,882 | <,001 | ,074 |

**Supplementary file: Table 5 Global functioning and quality of life MANOVA**

| **Table 5 a Multivariate Tests^a^** | | | | | | | |
| --- | --- | --- | --- | --- | --- | --- | --- |
| Effect | | Value | F | Hypothesis df | Error df | Sig. | Partial Eta Squared |
| CS vs TS | Pillai's Trace | ,317 | 20,298^b^ | 7,000 | 306,000 | <,001 | ,317 |
|  | Wilks' Lambda | ,683 | 20,298^b^ | 7,000 | 306,000 | <,001 | ,317 |
|  | Hotelling's Trace | ,464 | 20,298^b^ | 7,000 | 306,000 | <,001 | ,317 |
|  | Roy's Largest Root | ,464 | 20,298^b^ | 7,000 | 306,000 | <,001 | ,317 |
| a. Design: Intercept + DN0extreme1 | | | | | | | |
| b. Exact statistic | | | | | | | |

| **Table 5 b Tests of Between-Subjects Effects** | | | | | | | |
| --- | --- | --- | --- | --- | --- | --- | --- |
| Source | Dependent Variable | Type III Sum of Squares | df | Mean Square | F | Sig. | Partial Eta Squared |
| CS vs TS | GFS | 4310,663 | 1 | 4310,663 | 123,557 | <,001 | ,284 |
|  | EQ 5D 3L 1 | ,416 | 1 | ,416 | 1,954 | ,163 | ,006 |
|  | EQ 5D 3L 2 | 2,705 | 1 | 2,705 | 15,674 | <,001 | ,048 |
|  | EQ 5D 3L 3 | ,504 | 1 | ,504 | 2,156 | ,143 | ,007 |
|  | EQ 5D 3L 4 | ,146 | 1 | ,146 | ,371 | ,543 | ,001 |
|  | EQ 5D 3L 5 | 1,286 | 1 | 1,286 | 4,604 | ,033 | ,015 |
|  | EQ 5D 3L VAS | 2815,839 | 1 | 2815,839 | 7,764 | ,006 | ,024 |
